# Chimeric Antigen Receptor (CAR) T Cell Therapy in Acute Myeloid Leukemia (AML)

**DOI:** 10.3390/jcm8020200

**Published:** 2019-02-06

**Authors:** Susanne Hofmann, Maria-Luisa Schubert, Lei Wang, Bailin He, Brigitte Neuber, Peter Dreger, Carsten Müller-Tidow, Michael Schmitt

**Affiliations:** 1 Department of Internal Medicine V (Hematology/Oncology/Rheumatology), University Hospital Heidelberg, 69120 Heidelberg, Germany; Maria-Luisa.Schubert@med.uni-heidelberg.de (M.-L.S.); Lei.Wang@med.uni-heidelberg.de (L.W.); hebailin1990@gmail.com (B.H.); Brigitte.Neuber@med.uni-heidelberg.de (B.N.); Peter.Dreger@med.uni-heidelberg.de (P.D.); Carsten.Mueller-Tidow@med.uni-heidelberg.de (C.M.-T.) Michael.Schmitt@med.uni-heidelberg.de (M.S.); 2 National Center for Tumor Diseases (NCT), 69120 Heidelberg, Germany

**Keywords:** AML, CAR T cell, immunotherapy

## Abstract

Despite high response rates after initial chemotherapy in patients with acute myeloid leukemia (AML), relapses occur frequently, resulting in a five-year-survival by <30% of the patients. Hitherto, allogeneic hemotopoietic stem cell transplantation (allo-HSCT) is the best curative treatment option in intermediate and high risk AML. It is the proof-of-concept for T cell-based immunotherapies in AML based on the graft-versus-leukemia (GvL)-effect, but it also bears the risk of graft-versus-host disease. CD19-targeting therapies employing chimeric antigen receptor (CAR) T cells are a breakthrough in cancer therapy. A similar approach for myeloid malignancies is highly desirable. This article gives an overview on the state-of-the art of preclinical and clinical studies on suitable target antigens for CAR T cell therapy in AML patients.

## 1. Introduction

With conventional chemotherapy employing anthracycline and cytarabine, high complete remission (CR) rates of 60% to 80% in younger adults and 40% to 60% in older adults (>60 years) can be achieved [1,2]. Despite these successful response rates, relapse after conventional therapy is common, mainly due to the chemorefractoriness of leukemic stem cells [3,4]. The estimated five-year survival of acute myeloid leukemia (AML) patients in the years 2008–2014 was 27.4% [5]. Until now, allogeneic hemotopoietic stem cell transplantation (allo-HSCT) was the best curative treatment option in intermediate and high risk AML. However, allo-HSCT is not suitable for every patient and bears the risk of non-relapse mortality as well as relapse. Allo-HSCT and donor lymphocyte infusion (DLI) also suggest that cellular immunotherapy is effective in AML. Both allo-HSCT and DLI bear curative potential based on the graft-versus-leukemia (GvL) effect but endow the danger of life-threatening graft-versus-host disease (GvHD). The remaining challenge is to separate GvL from GvHD and to find ways to enhance GvL without inducing GvHD. This underlines the urgent need for novel effective treatment options that mediate enduring eradication of the leukemic tumor burden including leukemic stem cells (LSCs). 

Fueled by the success of immunotherapeutic strategies in other malignant hematologic entities, e.g., the anti-CD20 antibody rituximab in Non-Hodgkin’s-lymphoma (NHL) or the CD19-specific chimeric antigen receptor (CAR)-T-cell therapies in acute lymphoblastic leukemia (ALL) and NHL, several efforts have been made to develop antibody-based or cellular immunotherapies for AML. 

The key for successful targeted immunotherapies, either in form of an antibody or a targeted cellular approach, is the identification of a suitable target antigen. Cheever et al. summarized the features of an ideal target antigen, namely having a potential to induce clinical effects, being immunogenic, and playing a critical role in cell differentiation and proliferation of the malignant cells. Its expression should be restricted to malignant cells; it should be expressed in all malignant cells including malignant stem cells. A high number of patients should test positive for the antigen. The antigen should comprise multiple antigenic epitopes and be on the surface of malignant cells [6].

While for ALL, several other approaches, like bispecific antibodies and CAR-T-cells targeting CD19, are already in clinical practice, for AML identification of a good target antigen is more difficult. It is known from patients treated with rituximab that it is possible to live for some time with few B-cells, given the option that immunoglobulins can be substituted. Expression of antigens by AML blasts and leukemic stem cells is not exclusively restricted to those cells but overlaps with normal hematopoiesis, which can cause severe hematotoxicity of antigen-targeting therapies. 

The following paragraphs focus on CAR-T cell approaches in AML.

## 2. Adoptive Cellular Therapies

Based on the finding that cytotoxic T cells are key players in mediating GvL in allo-HSCT, concepts of adoptive T cell therapy were initially developed, such as tumor-infiltrating lymphocytes or donor lymphocyte infusion (DLI) [7,8,9]. Later, genetically engineered T cells were tested in clinical trials. Two main technologies of genetically engineered T cells exist—T cell receptor (TCR) engineered T cells and chimeric antigen receptor (CAR) transduced T cells. 

**Figure 1:**
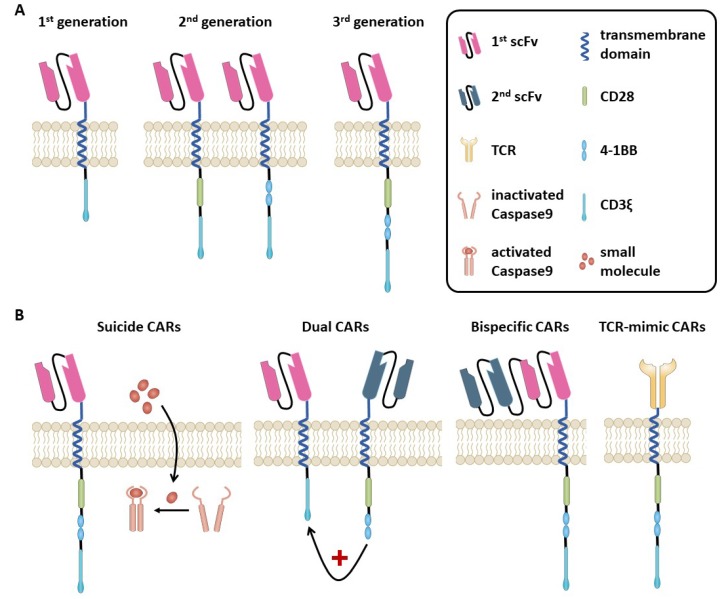
(**A**) Chimeric antigen receptor (CAR). CARs consist of an extracellular domain generated by joining the heavy and light chain variable regions of a monoclonal antibody with a linker to form a single-chain Fv (scFv) molecule. The antigen-specific domain binds its antigen on the surface of target cells. The scFv is attached via a hinge region to the transmembrane and intracellular receptor portion. In first-generation CARs, the signaling domain is composed of the zeta- domain of a T cell receptor (TCR)/CD3 receptor complex. In second- and third-generation CARs, one or two costimulatory signaling domains are added (e.g., CD28, 4-1BB (CD137), OX-40 (CD137), or inducible T cell costimulatory (ICOS)) within their intracellular domain, respectively. (**B**) Innovative CAR design. Suicide gene strategies are investigated as control mechanisms for better toxicity management of CAR T cells. One example is the inducible caspase 9 (iCasp9). When the small molecule AP1903 is administered, iCasp9 domains dimerize and activate apoptosis independently of CAR activation. Dual-targeting CARs express two different antigen-specific CARs, whereas bispecific CARs bear two linked scFV within one CAR construct. To address human leukocyte antigen (HLA)-presented antigens, TCR-mimic (TCRm) CARs directing the scFv domain against a peptide-HLA complex have been developed.

Both approaches directly place the T cell in the vicinity to the antigen-bearing target cell. One main difference is that a T cell receptor (TCR) recognizes intracellularly and extracelluarly expressed antigens in the context of human leukocyte antigen (HLA)- receptors, whereas CAR T cells are HLA-independent and only recognize surface antigens in an antibody-specific manner (Figure 1).

CAR T-cells combine the strong feature of an antibody in target recognition and the effector, and long-term function of the T-cell and the effector cell is directly brought to the cancer cell. CARs (Figure 1) are artificial receptors composed of three domains, (1) an extracellular antigen-specific binding domain that is derived from an antibody’s single chain variable fragment (scFv), (2) a hinge and transmembrane segment usually derived from CD8alpha [10] or IgG domain [11], and (3) an intracellular T-cell signaling domain.

CAR T cells are genetically engineered to express CARs via viral (retroviral, lentiviral, adenoviral) or non-viral technologies such as electroporation, transposon-based, or gene-editing systems. 

Addition of co-stimulation signals to the intracellular domain in second and third generation CARs aim to improve the survival of engineered T cells (Figure 1A). First-generation CARs contain only the tyrosine-based zeta-signal-transducing subunit from the TCR/CD3 receptor complex [12,13,14]. Adjacent to this zeta-domain, second-generation CARs harbor one and third-generation CARs two additional costimulatory molecules [15] such as CD28 [16], CD27, DAP-12 [17], 10 4-1BB (CD137), OX40 (CD134) [18], or inducible T cell costimulator (ICOS) [19]. Indeed, depending on the introduced costimulatory signal, second and third generation CARs mediate superior activation, proliferation, and in vivo persistence of T cells [20,21]. Third generation CARs show increased tumor-lytic activity as well as reduced activation-induced cell death compared to first-generation CARs [22,23]. 

When relapse occurs after antibody or CAR therapy, tumor cells often lose the targeted antigen. This problem is addressed by CAR T cells targeting multiple antigens, either by simultaneous co-administration of several monospecific CARs [24] or by one distinct CAR T cell targeting several antigens (Figure 1B). These CAR T cells are called dual-targeting T cells (when one CAR T cell expresses two different antigen-specific CARs [25]) or bispecific CAR T cells (when one CAR is specific for two different targets [26]). This combinatorial CAR therapy approach was recently put forward by Perna et al. With the help of high-throughput surfaceome expression data, they identified pairs of target antigens and defined ideal features of CAR targets to reduce the risks of antigen escape and off-tumor toxicity. The features for an ideal pair are: no overlapping expression in normal tissues to minimize systemic off-tumor toxicity, very low level expression in CD34+CD38- hematopoietic stem cells (HSCs) to minimize cytotoxicity, very low expression in normal resting and activated T cells to minimize T cell reactivity, expression (for the combination) in all tumor cells to overcome clonal heterogeneity, expression in LSCs, and co-expression in tumor-cells to prevent antigen escape [27]. Besides antigen escape, loss of CAR T cells and autoantibody development are important mechanisms of CAR T cell therapy failure [28].

**Figure 2:**
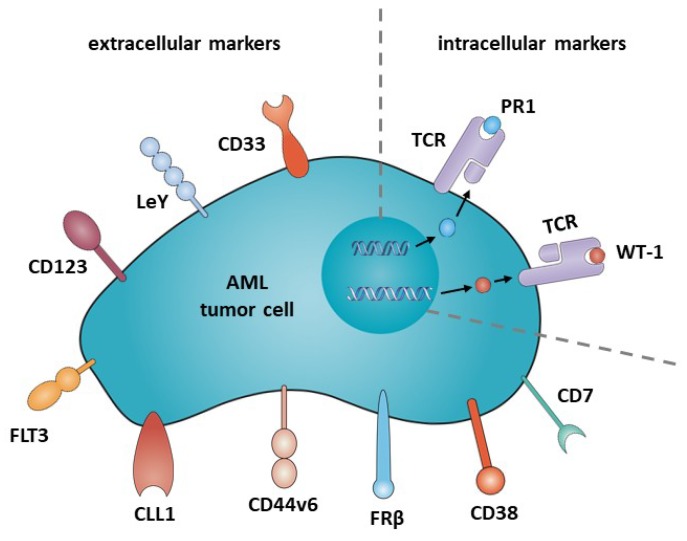
Potential target antigens for CAR therapy in AML.

To date, in the context of AML, only few CARs have been investigated in clinical trials, and in contrast to B-cell malignancies, no licensing authority approved CAR therapy for AML exists. In the following sections, we give an overview of antigen candidates that are already investigated in clinical trials (Table 1), as well as those that are potentially suitable for CAR therapy in AML (Figure 2). 

## 3. CD33

CD33 is expressed in up to 90% of leukemic blast cells but also on healthy myeloid and myeloid progenitor cells [30,31]. It is not expressed on early pluripotent CD34-positive hematopoietic stem cells [32], but it is also expressed by hepatocytes, which can explain extrahematological toxicity in the form of veno-occlusive liver disease (VOD) [33,34]. A restriction is that CD34+CD33- negative leukemic stem cells have been reported [35]. CD33 is an attractive target for immunotherapy against AML. This was shown by the development of Gemtuzumab (Mylotarg®, Pfizer, Berlin, Germany), a humanized drug-conjugated anti-CD33-antibody. Although first approved in 2000 by the US Food and Drug Administration (FDA), it was withdrawn from the European and US markets in 2010 due to bone-marrow toxicity and VOD. It was reintroduced in 2018 after a meta-analysis by Hills et al. demonstrated that a low, fractionated dose of Mylotarg® in combination with chemotherapy led to an improved overall-survival of 280 treated AML patients [36]. 

Due to this experiment and the high expression of CD33 in myeloid leukemia, there are currently many activities considering anti-CD33 CAR therapy (Table 1). To date, one report of a patient with r/r AML who was treated with anti-CD33 CARs has been published [37]. In this phase I trial, the patient received a total of 1.12 × 10^9^ autologous T-cells (38% CAR transduced) and suffered from cytokine release syndrome (CRS) as well as pancytopenia and disease progression nine weeks after cell infusion. 

Due to the CD33 expression in healthy myelopoiesis, it is necessary to develop new safety concepts with anti-CD33 CAR transfusion.

One approach is the transient expression of anti-CD33 CAR, which was tested in an in vivo model of AML-xenotransplanted NOD scid gamma (NSG) mice [38]. Only transient cytotoxicity was observed. Another interesting method recently published is the generation of leukemia specificity by genetic knock-out of CD33 in normal hematopoietic stem and progenitor cells. Thereby, an artificial resistance against anti-CD33 CAR T cell therapy is created. In xenograft immunodeficient mice, CD33-deficient human HSPCs engrafted and differentiated normally. In rhesus macaques, anti-CD33 CAR T cell therapy transfused after autologous CD33 knock-out HSPC transplantation was effective in eliminating leukemia cells without any signs of myelotoxicity [39]. 

## 4. Lewis Y (LeY)

Lewis Y (LeY) is a carbohydrate antigen that is overexpressed by a wide variety of epithelial cancers [40] and hematological malignancies including AML [41,42] but with only limited expression on normal tissue [43]. 

In 2010, Peinert and colleagues published the results of the first phase I CAR- T cell trial for relapsed LeY-expressing AML [44]. They investigated an autologous second-generation anti-LeY CAR in four patients who received up to 1.3 × 10^9^ total T cells (14–38% with anti-LeY CAR expression). No grade three or four toxicity was observed. The best response was transient cytogenetic remission in one patient; another patient showed a transient reduction of blasts, and two patients showed stable disease. All patients relapsed after 28 days to 23 months after adoptive cell therapy. CAR T cell persistence was demonstrated for up to 10 months. 

## 5. CD123 

CD123 is the transmembrane alpha chain of the interleukin 3 receptor. Due to its surface expression and its overexpression on AML blasts and LSCs, as well as its low expression on normal hematopoeietic stem cells, CD123 qualifies as a suitable target [45,46]. However, similar to CD33 targeted therapy, the problem of myelotoxicity in CD123 targeted therapy remains.

At the time of writing this manuscript, CD123 is being studied in 11 clinical trials for AML (Table 1). 

CARs normally encode in its scFvs a VH and VL chain from one monoclonal antibody in the extracellular antigen binding domain. In an experimental AML model, hematopoietic toxicity was shown after treatment with anti-CD123 CAR T cells. When using VH and VL chains derived from different CD123-specific mAbs for CAR engineering, one specific combination showed less lysis of the normal hematopoietic stem cells while preserving the toxicity [47]. 

## 6. FLT3 (CD135)

FLT3-ITD mutations are found in about 20% (and FLT3-TKD in about 7%) of all AML patients [48]. In a preclinical model, second-generation anti-FLT3-41BB CARs were tested [49]. Specific cytotoxicity against FLT3+ leukemia cell lines and primary cell lines in vitro, as well as little off-tumor cytotoxicity on normal hematopoietic stem cells, was observed. In a xenograft mouse model, prolonged survival was seen in FLT3+ mice that were treated with the anti-FLT3 CARs. Compared to anti-CD33 CAR T cells, less toxicity to hematopoietic stem cells and multipotent myeloid progenitor cells and equivalent toxicity to common myeloid progenitor and granulocyte-macrophage progenitor cells was described, suggesting a lower hematologic toxicity with anti-FLT3 CAR T cells. In a second preclinical study, second-generation 4-1BB CARs that target the FLT3-ligand (FLT3L) were tested [50]. For anti-FLT3L, little off-tumor cytotoxicity on normal hematopoietic stem and progenitor cells was observed. A xenograft mouse model also showed a significantly prolonged survival in FLT3+ leukemia bearing mice after anti-FLT3L CAR T cells [50]. 

## 7. CLL1

The myeloid surface antigen C-type lectin-like molecule 1 (CLL1 or CLEC12A) is a glycoprotein highly expressed by the majority of AML patients. It is expressed on AML blasts and on normally differentiated myeloid cells. Relatively low amounts are expressed on CD34+ progenitor cells. It is not expressed on normal hematopoietic stem cells [51]. CLL 1 therefore qualifies as a promising CAR T cell target suggesting low “off-tumor” toxicity. 

Four research groups generated anti-CLL1 CAR T cells, three second generation [52,53,54] and one third generation CAR [55]. All four showed potent activity against CLL1+ AML cell lines, as well as primary CLL1+ AML blasts in vitro and in xenograft mouse models, while sparing normal myeloid precursor cells. Tashiro et al. went a step further and introduced the inducible caspase-9 suicide gene system into the CARs and could successfully control anti-CLL1 CAR T cell activity in vitro and in vivo. 

## 8. CD44v6

CD44v6 is the isoform variant 6 of the hyaluronic acid receptor CD44, a class I membrane glycoprotein overexpressed in hematologic malignancies including AML [56] and epithelial tumors [57]. It is absent in hematopoietic stem cells [58] and shows low expression levels on normal cells. Casucci and colleagues designed a second generation anti-CD44v6 CAR with cytotoxicity against AML cells while sparing normal hematopoietic stem cells [59]. Monocytopenia was the dose limiting toxicity in this preclinical study. To control this adverse event, the clinical-grade suicide genes, thymidine kinase [60] and the nonimmunogenic inducible Caspase 9 (iC9) [61], were coexpressed in the anti-CD44v6 CARs with iC9 successfully eradicating the CAR T cells within hours. 

## 9. Folate Receptor ß (FRß)

Folate receptor ß (FRß) is primarily expressed on myeloid-lineage hematopoietic cells [62] and is expressed on about 70% of primary AML cells [63]. The expression of FRß on AML blasts can be increased by all-trans retinoic acid (ATRA) and enhanced the efficacy of folate-conjugated drug therapy in a preclinical study [64,65]. Preclinical models showed the efficacy of anti-FRß CAR T cells and an even better efficacy of high-affinity anti-FRß CAR T cells against AML cells in vitro and in vivo without toxicity against healthy hematopoietic progenitor/stem cells (HPSCs) [66,67].

## 10. CD38

CD38 is expressed on the majority of AML blasts but not healthy human hematopoietic stem cells [68,69]. Due to the modest expression level of CD38 in AML, the combination of ATRA and second generation anti-CD38 CAR T cells to enhance the CD38 expression was tested [70]. In this study, ATRA enhanced the cytotoxicity of anti-CD38 CAR T cells on AML cells with the augmented CD38 expression in vitro.

## 11. CD7

CD7 is expressed by T cells and natural killer cells [71]; it is also expressed in over 90% of lymphoblastic T cell leukemia and lymphoma [72,73] and in about 30% of AML cases [74,75], but is absent in normal myeloid and erythroid cells. An anti-CD7 CAR in CD7 knock-out T cells to prevent fratricide can effectively eliminate CD7 + AML cell lines as well as primary AML cells while sparing normal myeloid and erythroid progenitor cells [76,77,78]. 

## 12. Intracellular Targets: PR1/HLA-A2; WT1/HLA-A2

The majority of leukemia-associated-antigens and neoantigens are intracellularly processed and presented by HLA class II molecules. To address HLA-presented antigens, TCR-mimic (TCRm) CARs directing the scFv domain against a peptide-HLA complex were developed. Proteinase 1 (PR1) is a HLA A2-restricted nonamer derived from the leukemia associated antigen proteinase 3 and neutrophil elastase. Both proteases are expressed in the primary azurophilic granules of neutrophils and are overexpressed in myeloid leukemic blasts [79,80]. A second-generation CAR construct targeting HLA-A2/PR1 was preferentially cytotoxic against human AML cell lines and primary AML blasts in vitro [81]. The second TCRm CAR published targets the leukemia associated antigen Wilms tumor 1 (WT1) in the context of HLA-A2 and has demonstrated efficacy in vivo in an AML mouse model [82]. WT1 is overexpressed in AML, chronic myeloid leukemia (CML), and several solid tumors [83,84,85]. Another antigen candidate is PRAME (preferentially expressed antigen in melanoma). It is a so-called cancer-testis antigen and is therefore exclusively expressed in the testes and ovaries in healthy tissue. However, in several malignant tissues—and in about 20–40% of AML cases—it is intracellularly expressed and presented on the cell surface via human leukocyte antigen (HLA)-I. Chang et al. developed a TCRm human IgG1 antibody that recognizes a decamer peptide derived from PRAME in the context with HLA-A. It showed therapeutical effectiveness against mouse xenograft models of human leukemia [86]. In addition, a multicenter phase I/II clinical trial is currently testing autologous T cells that are transduced with a PRAME-specific HLA-A*02:01-restricted TCR (NCT03503968). 

Whether autoimmune reactions as off-tumor toxicity occur with TCRm CAR application has to be investigated in further studies. 

## 13. Safety Affairs

Relevant side effects of CARs are tumor-lysis syndrome and cytokine release syndrome, as well as “on-target but off-tumor” toxicity. “On-target but off-tumor” toxicity occurs when the target antigen is not only expressed on the target cells but also on normal tissues. This is the case for HER2, which is expressed in epithelial cells in the gastrointestinal, respiratory, reproductive, urinary tract, skin, breast, placenta, and normal hematopoietic cells [87]. A clinical trial investigating a third generation CAR targeting HER2 reported of one patient who developed respiratory distress within 15 min after receiving a single dose of 10^10 CAR T cells, followed by cardiac arrest [88]. This study underlines how important the target antigen selection is. Other aspects of reducing toxicity, mainly cytokine storm, are the number of infused CARs as well as the use of immunosuppressive agents and an introduced control mechanism into the CARs (Figure 1B). As control mechanisms, several suicide gene strategies were investigated, including thymidine kinase gene of the herpes simplex virus thymidine kinase (HSV-TK) [89] and the inducible caspase 9 (iCasp9) [90]. An elegant approach to limit “off-tumor” toxicity is to modify the CAR scFvs affinity of the antibody [91]. For high affinity HER2 CAR T cells, it was demonstrated that in dependence of the antigen density on the surface of the target cell, a high-affinity CAR is reactive against a malignant (high density) but not a normal (low density) cell [91]. 

## 14. Conclusions

Although in B-cell malignancies, CAR T cells now begin to build one therapeutic column in clinical practice, the value of CAR T cell therapy for AML still has to be determined. 

There are major hurdles to take, e.g., finding the right antigen with low “off-tumor” toxicity and supplementing strategies to minimize “off-tumor” toxicity. Several attempts have already been made, such as suicidal control of CAR T cells, temporary expression of the CAR, and improvement of the affinity of the CAR. The CAR race has started and will hopefully improve and enrich the therapeutic armentarium against AML.

## Figures and Tables

**Table 1:** Chimeric antigen receptor (CAR) trials in acute myeloid leukemia (AML) [29].

Trial ID	Status	Phase	Target	Indication	Institution
NCT03585517	R	I	CD123	CD123+ AML	Xian Lu, Beijing, China
NCT03114670	R	I	CD123	recurred AML after allo	Fengtai District, Beijing Shi, China
NCT03556982	R	I/II	CD123	R/R AML	307 Hospital of PLA, Beijing, Beijing, China
NCT02623582	terminated	I	CD123	R/R AML	Abramson Cancer Center of the University of Pennsylvania, Philadelphia, Pennsylvania, United States
NCT02159495	R	I	CD123	R/R AML, Persistent/Recurrent Blastic Plasmacytoid Dendritic Cell Neoplasm	City of Hope Medical Center, Duarte, California, United States
NCT03672851	R	I	CD123	R/R AML	Second Affiliated Hospital of Xi’an Jiaotong University, Xi’an, Shaanxi, China
NCT03766126	R	I	CD123	R/R AML	University of Pennsylvania, Philadelphia, Pennsylvania, United States
NCT01864902	R	I	UCART 123	R/R AML, newly diagnosed high-risk AML	Weill Cornell Medical College, New York, New York, United States MD Anderson Cancer Center, Houston, Texas, United States
NCT03631576	R	II/III	CD123/CLL1	R/R AML	Fujian Medical University Union Hospital, Fuzhou, Fujian, China
NCT03126864	R	I	CD33	R/R CD33+ AML	University of Texas MD Anderson Cancer Center, Houston, Texas, United States
NCT02799680	unknown	I	CD33	R/R AML	Affiliated Hospital of Academy of Military Medical Sciences, Beijing, Beijing, China| Chinese PLA General Hospital, Beijing, Beijing, China
NCT01864902	unknown	I/II	CD33	R/R AML	Biotherapeutic Department and Pediatrics Department of Chinese PLA General Hospital, Hematological Department, Affiliated Hospital of Changzhi Medical College, Beijing, Beijing, China
NCT02944162	unknown	I/II	anti-CD33 NK CAR	R/R CD33+ AML	PersonGen BioTherapeutics (Suzhou) Co., Ltd., Suzhou, Jiangsu, China
NCT03291444	R	I	CD33, CD38 CD56, CD117, CD123, CD34, and Muc1 for AML and MDS	R/R AML, MDS; ALL	Zhujiang Hospital, Southern Medical University, Guangzhou, Guangdong, China
NCT03473457	R	n.a.	single CAR-T or double CAR-T cells with CD33, CD38, CD56, CD123, CD117, CD133, CD34, or Mucl	R/R AML	Southern Medical University Zhujiang Hospital, Guangdong, Guangdong, China
NCT03222674	R	I/II	Muc1/CLL1/CD33/CD38/CD56/CD123	AML	Zhujiang Hospital of Southern Medical University, Guangzhou, Guangdong, China| Shenzhen Geno-immune Medical Institute, Shenzhen, Guangdong, China| Yunnan Cancer Hospital & The Third Affiliated Hospital of Kunming Medical University & Yunnan Cancer Center, KunMing, Yunnan, China
NCT02203825	completed	I	NKG2D	AML, MDS-RAEB, and Multiple Myeloma.	Dana-Farber Cancer Institute, Boston, Massachusetts, United States
NCT03018405	R	I/II	NKR2 (NKG2D)	R/R AML, AML, Myeloma	Tampa, Florida, United States| Buffalo, New York, United States| Brussels, Belgium|Brussels, Belgium| Ghent, Belgium
NCT03018405	unknown	I/II	CD7/NK92 cell	CD7+ R/R Leukemia and Lymphoma	PersonGen BioTherapeutics (Suzhou) Co., Ltd., Suzhou, Jiangsu, China
NCT01716364	unknown	I	Lewis Y	Myeloma, AML, MDS	Peter MacCallum Cancer Centre, Melbourne, Victoria, Australia

Abbreviations: R, recruiting; r/r relapsed/refractory; AML, acute myeloid leukemia; ALL, acute lymphoblastic leukemia; MDS, myelodysplastic syndrome. Note: Search term: CAR, AML (as by 12 December 2018). Source: [29].
